# Anillin Recedes in p53-Dependent Senescence of Tumor Cells and Reappears in Cells Escaping from Senescence

**DOI:** 10.14336/AD.2025.0402

**Published:** 2026-05-10

**Authors:** Tomasz Buko, Karolina Staniak, Magdalena Dudkowska, Dorota Janiszewska, Dominika Dębowska, Agnieszka Gadecka, Anna Bielak-Zmijewska

**Affiliations:** ^1^Laboratory of Molecular Basis of Aging, Nencki Institute of Experimental Biology, Polish Academy of Sciences, 02-093 Warsaw, Poland; ^2^Laboratory of Calcium Binding Proteins, Nencki Institute of Experimental Biology, Polish Academy of Sciences, 02-093 Warsaw, Poland; ^3^Laboratory of Biomolecular Interactions Studies, Chair of Drug and Cosmetics Biotechnology, Faculty of Chemistry, Warsaw University of Technology, 00-664 Warsaw, Poland; ^4^Laboratory of Cytometry, Nencki Institute of Experimental Biology, Polish Academy of Sciences, 02-093 Warsaw, Poland

**Keywords:** anillin, p53, senescence, tumor cells, cells escaping senescence

## Abstract

Anillin is a protein whose most recognizable function is coordinating the spatial distribution of cytoskeletal proteins during the course of cytokinesis. Its level increases in many types of cancer, and therefore, it has been proposed as a prognostic marker for some of them. Anillin is detected in the cell nucleus but so far, its nuclear role has not been recognized. Our recent studies have shown that anillin gene expression and protein level decrease in normal senescent cells, which could be attributed to inhibition of proliferation. However, its presence in the nucleus of neurons, postmitotic cells, suggests functions other than regulation of cytokinesis. Some data indicate that anillin expression may be regulated by p53, a protein usually induced in cells undergoing senescence due to various stimuli. The presence of p53 binding sites in the upstream promoter region of the anillin gene was mainly shown in *in silico* studies, and, so far, few experimental data seem to confirm such regulation. This study aimed to test whether anillin level decreases in senescent cancer cells and whether this is due to regulation by p53. Using p53-proficient and p53-deficient cancer cells, we have shown that anillin levels decreased in cells whose senescence was p53-dependent. We also showed that in cells escaping senescence, anillin reappeared with proliferation resumption, which correlated with decreased p53 levels. Our results demonstrate the universality of anillin reduction during the course of senescence and show that p53 is a negative regulator of this protein.

## INTRODUCTION

Anillin is a highly evolutionarily conserved protein regulating cell division [1-3]. The multi-domain structure of anillin allows it to act as a scaffold for numerous cytoskeletal proteins, including those involved in cytokinesis [4]. During mitosis, anillin is involved in the formation of the division furrow by the positioning and crosslinking proteins such as actin, myosin, septin and RhoA, which together determine daughter cell separation [5]. Another recognized anillin function is regulation of the formation of tight and adherent junctions, which are critical for the maintenance of the epithelial barrier [6-8]. During interphase, anillin accumulates mainly in cell nucleus targeted by importin β2 in a Ran-dependent manner [9-10], but there is no information on its role there. The nuclear localization could be related to a yet unknown nuclear function or essential to prevent cytosolic accumulation of anillin, which could negatively affect the cell’s architecture. In this regard, it has been shown that nuclear localization signal (NLS) depletion in anillin caused actin cytoskeleton reorganization. One opinion posits that nuclear accumulation during interphase serves to build a reservoir of anillin for the subsequent cell division. The presence of anillin in the nucleus of postmitotic cells contradicts this opinion [11]. Nuclear anillin could be involved in chromatin organization, transcriptional regulation and DNA damage repair [12]; however, more experimental data are necessary to prove that. Anillin may be involved in the regulation of gene expression by binding to transcription factors such as CDC5L, TAF10, Myc and BRCA1 [12]. As anillin possesses an actin-binding domain its role in regulating gene expression may also result from its interaction with nuclear actin [2]. While the function of anillin in the nucleus remains a mystery, the role of nuclear actin is relatively well understood. Nuclear actin regulates transcription (interacts with RNA polymerase), including transcription of rDNA, chromatin remodeling, intranuclear movement and migration of interphase chromosomes. It also controls chromatin accessibility, organization (it is a constituent of chromatin remodeling complexes such as BAF and SWR1, which affect the conversion of heterochromatin to euchromatin), gene expression and is involved in DNA repair [13-16]. Therefore, it cannot be ruled out that anillin, through its interaction with actin, regulates similar processes in the nucleus.

One of the most intensively studied aspects of anillin function is its association with tumorigenesis [11, 17-19]. Compared to normal cells, increased anillin expression was detected in many types of cancer, such as breast, gastric, lung, pancreatic and melanoma [9, 20]. Increased anillin level is associated with more invasive cancer metastasis and a poorer patient prognosis [20-22]. Anillin is involved in cell migration and invasion [12]. On the other hand, the knockdown of anillin in mice hepatocytes reduced the development of liver tumors [23].

Anillin gene expression is cell cycle-dependent [24]. Recent studies suggest that anillin expression could be negatively regulated by p53, a protein recognized as a central regulator of cellular senescence [25]. Bioinformatics prediction and chromatin immune-precipitation analysis showed four potential p53 binding sites in the upstream region of anillin gene promoter [20, 26, 27]. Differences in anillin levels were observed in p53-deficient and p53-proficient colon cancer HCT116 cells after induction of DNA damage [20]. In cells lacking p53, anillin level did not change, contrary to p53-proficient cells, in which a diminished level of anillin coincided with an increased level of p53. Up to now, changes in the anillin level have not been analyzed in cells undergoing senescence, and its depletion was attributed only to DNA damage. p53 is mutated (unfunctional) in many types of tumors, and it was shown that the anillin level is upregulated in several cancer types. However, there are no data showing if tumor cells carrying a wild-type copy of the gene encoding p53 have lower levels of anillin than cells carrying a mutation in this gene. Bioinformatics studies determined which genes were co-expressed with anillin in malignant tumor cells. The analyses identified several proteins involved in cell division, cell cycle regulation, and, interestingly, the senescence process [27].

Cellular senescence is one of the fundamental mechanisms responsible for inhibiting cancer growth, and initial reports of therapy-induced senescence (TIS) of cancer cells gave high hopes for the treatment of this group of diseases [28, 29]. However, it appeared that TIS can lead to a resumption of cancer cell divisions [30]. A unique feature of senescent cancer cells is the ability to polyploidize, which may contribute to the escape from the senescence pathway, as was observed in colon cancer cells (HCT116 p53WT) treated with doxorubicin [31, 32].

This study aimed to determine if anillin level declines during cancer cell senescence and to verify a possible negative regulation of anillin expression by p53. Our results have shown that cancer cells subjected to doxorubicin-induced senescence downregulated anillin levels but only those that were p53-proficient. This was associated with an increase in p53 levels, which supports the tested hypothesis. On the other hand, in doxorubicin-treated p53-deficient cells, which did not express typical senescence markers observed in p53-proficient cells, the level of anillin decreased slightly. Data concerning cells escaping from senescence showed that anillin gradually reappeared upon resumption of proliferation, in inverse relation to the gradual decline of p53. In summary, we propose anillin as a universal marker of cells undergoing p53-dependent senescence, and p53 is proposed as a negative regulator of anillin.

## MATERIALS AND METHODS

### Cell culture

**Figure 1.**
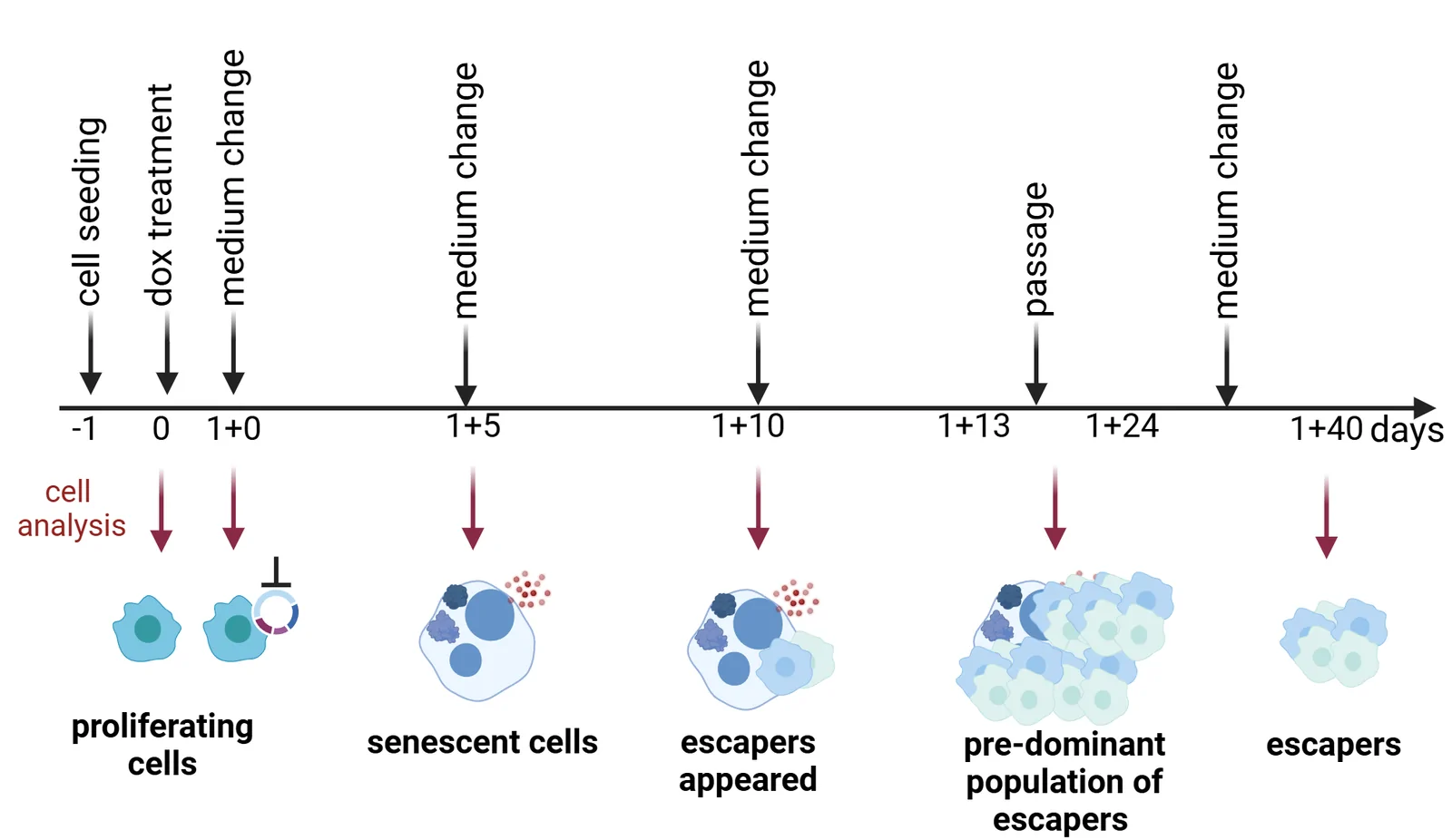
Scheme of cell treatment with doxorubicin and cell escapers analysis.

The human HCT116 p53-proficient (referred to as HCT116 p53WT) colon cancer cell line and the breast cancer cell line MCF-7 were obtained from ATCC (HCT116 CCL-247; MCF-7 HTB-22). The human HCT116 p53-deficient (HCT116 p53KO) colon cancer cell line was kindly provided by Prof. Bert Vogelstein (Johns Hopkins University, Baltimore, MD, USA). Cells were grown under standard conditions (37°C, 5% CO_2_) in McCoy’s (HCT116; Biowest), or Dulbecco’s modified Eagle’s low glucose medium (MCF-7; Sigma-Aldrich), supplemented with 10% FBS (Biowest) and antibiotics (Sigma-Aldrich). For doxorubicin treatment, cells were seeded at a density of 5000/cm^2^. After 24 h of culture, doxorubicin (Sigma-Aldrich) was added to the cell medium for the next 24 h and then replaced with a fresh medium. The final concentration of doxorubicin was 100 nM in the case of HCT116 p53KO and MCF-7 and 300 nM for HCT116 p53WT (doxorubicin doses were selected for efficient senescence induction based on analysis of cell death, proliferation and SA-β-Galactosidase activity – see supplementary data (Supplementary Fig. 1, 2, 3, 4, 7)). Cells were collected after 24h of drug treatment (day 1) or on subsequent days of culture in fresh medium (day 1+N) corresponding to various phases of the senescence process as illustrated in Fig. 1 – day 1+0: post-treatment effect; day 1+5 – 1+7: senescence; day 1+10: first senescence escapers appeared; day 1+13 – 1+24: predominant population of senescence escapers; ESCAPERS: cells escaping senescence cultured for one month counting from day 1+13 – 1+24. Control cells were collected 24 hours after seeding.

We also tested if the effectiveness of senescence induction differed when cells were exposed to doxorubicin permanently for 5 days (marked as D1 – day 1, D5 – day 5) instead of 24 hours and then cultured in a fresh medium (Supplementary Fig. 3).

### Bromodeoxyuridine (BrdU) incorporation assay

The cell's capacity to replicate DNA was evaluated using the BrdU incorporation assay. BrdU (Becton Dickinson) was added to the culture medium at a concentration of 10 µM for 24 hours. Next, the cells were fixed in ice-cold 70% ethanol and stored at -20°C for at least 24 hours. The cells were then immunostained with a primary anti-BrdU antibody (Becton Dickinson, 347580, 1:100) followed by a secondary Alexa Fluor 488-conjugated antibody (ThermoFisher Scientific, A-11029, 1:500). DNA was counterstained with DAPI (1 µg/ml, Sigma-Aldrich). Cells were then analyzed using a Nikon Eclipse Ti fluorescent microscope. The percentage of dividing cells was determined by calculating the number of BrdU-positive cells relative to the number of all DAPI-stained nuclei. More than 100 cells from 10 randomly selected fields were counted per sample for each biological replication.

### Senescence-Associated β-Galactosidase (SA β-Gal) assay

Cells were stained following the method described by Dimri et al. [33]. After fixation (2% formaldehyde and 0.2% glutaraldehyde in PBS), the cells were washed and incubated in buffer (5 mM potassium ferrocyanide, 5 mM potassium ferricyanide, 150 mM NaCl, 2 mM MgCl_2_, 0.02 M phosphate buffer, and 1 mg/ml X-Gal, pH 6.0) overnight at 37°C in the dark and without carbon dioxide exposure. Nuclei were counterstained with DAPI (1 µg/ml). Cells were then analyzed using a Nikon Eclipse Ti fluorescent microscope. The results are presented as the number of cells with increased enzyme activity (blue color) relative to the total number of cells (based on DAPI-stained nuclei). More than 100 cells from 10 randomly selected fields were counted per sample for each biological replication.

### Western blotting

Whole-cell protein lysates were prepared in the Laemmli buffer [34]. An equal amount of total protein was electrophoretically separated in 10, 12 or 15% SDS-polyacrylamide gels (electrophoresis buffer: 25 mM Tris-HCl pH 8.3, 250 mM glycine, 0.1% SDS), transferred to nitrocellulose membrane (transfer buffer: 25 mM Tris-HCl pH 8.3, 200 mM glycine, 0.05% SDS, 20% methanol) which was blocked in 5% non-fat milk in TBS containing 0.1% Tween-20 for 1 hour in RT. Then, membranes were incubated overnight at 4°C with primary antibodies: anti-ANLN (Abcam, ab211872, 1:1000), anti-p53 (Santa Cruz, sc-126, 1:500), anti-GAPDH (Millipore, MAB374, 1:150000), anti-LMNB1, (Santa Cruz, sc-365962, 1:500), anti-HMGB1 (Abcam, ab79823, 1:500), anti-H3K9me3 (Diagenode, C15410193, 1:1000), washed and incubated for 1 hour in RT with horseradish peroxidase-conjugated secondary antibody (Dako, P0447 or P0448, 1:2000). The ECL system (ThermoFisher Scientific) was used according to the manufacturer’s instruction. Protein levels were normalized to GAPDH and shown as a relative fold change compared to the control.

### Immunofluorescence

Cells were cultured according to the experimental variant. Next, cells were fixed with 4% paraformaldehyde for 15 minutes and stored in 70% ethanol at -20°C for at least 24 hours. After washing in PBS, cells were permeabilized for 10 minutes in PBS with 0.5% Triton, blocked for 10 minutes in blocking buffer (PBS with 2% BSA, 1.5% goat serum and 0.5% Triton), and incubated for 2 hours with primary antibody specific for ANLN (Sigma, HPA050556, 1:250), B23 (Abcam, ab10530, 1:100) or LMNA/C (Cell Signaling, mAb#4777, 1:2000) dissolved in blocking buffer. Then, cells were washed and incubated for 1 hour with a secondary antibody conjugated with fluorescent AlexaFluor ® dye (ThermoFisher Scientific, A-11008 or A-11029, 1:500) dissolved in PBS. Nuclei were stained with DAPI (1 µg/ml). To validate antibody specificity, secondary antibody only control was employed. Finally, slides were mounted in Fluoromount G (Invitrogen) and scanned using an Eclipse Ti fluorescent microscope (Nikon) under 40× magnification. Computational analyses of cell fluorescence intensity were performed using ImageJ (FiJi) software. More than 100 cells from 20 randomly selected fields were counted per sample for each biological replicate.

### Statistical analysis

All experiments were performed in at least three biological replicates for all experimental conditions. Results are presented as a means with standard deviation unless stated otherwise. Normality was checked using Shapiro-Wilk test, and statistical analysis was performed using an appropriate test (t-Student test, Wilcoxon matched-pairs signed-rank test, Kruskal-Wallis test or one-way ANOVA test). Statistical analysis was performed using Prism 10.2.3 (GraphPad Software Inc, la Jolla, USA). The p-value is stated as: * p ≤ 0.05, ** p ≤ 0.01, *** p ≤ 0.001, **** p ≤ 0.0001. The number of biological replicates (n) and statistical test used for group analysis is denoted in all figure legends.

## RESULTS

### Reduction of anillin level is associated with senescence of tumor cells

**Figure 2.**
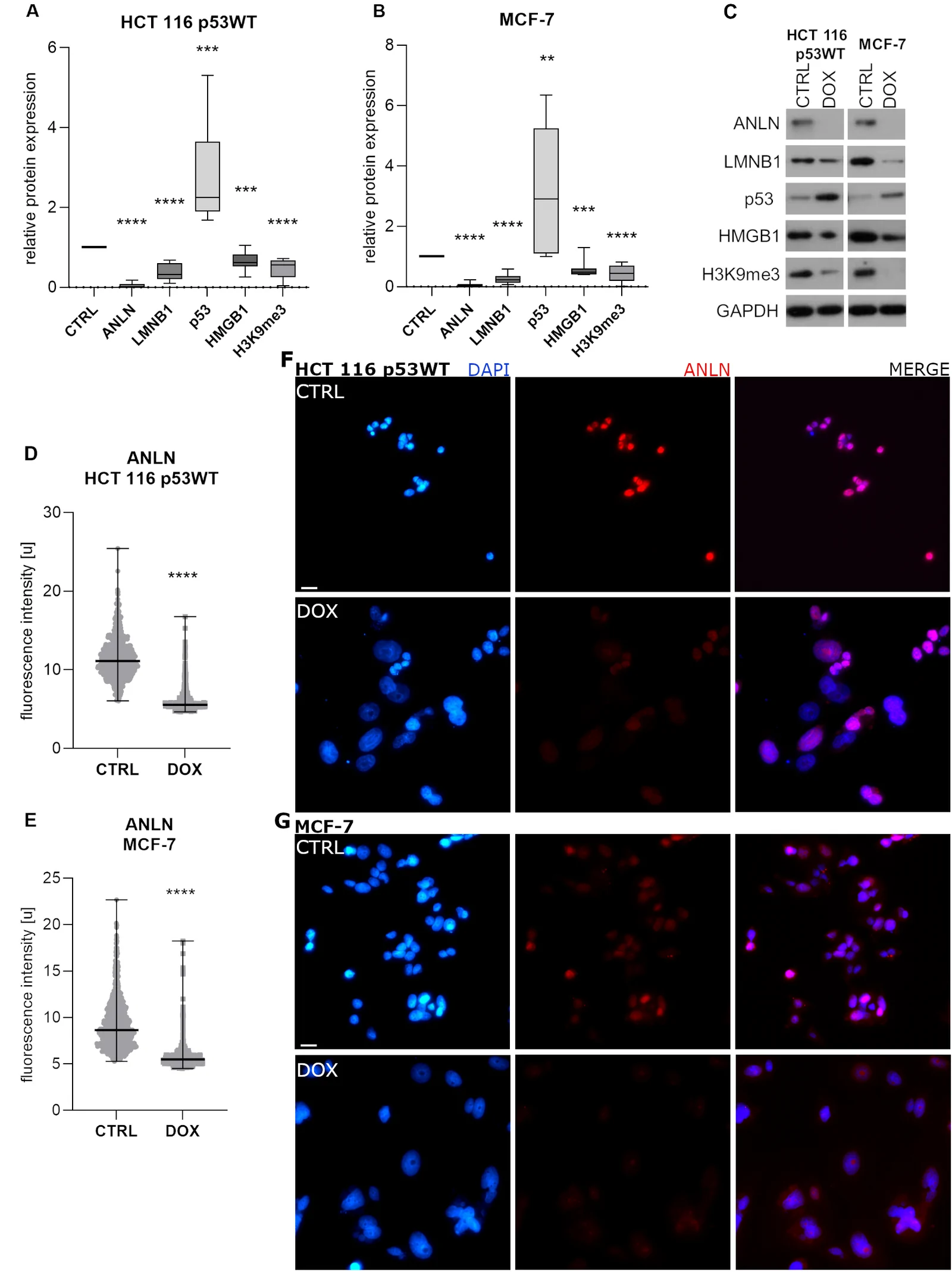
**Analysis of the selected senescence markers and anillin levels in HCT116 p53WT and MCF-7 cells induced to senesce by 1 day-treatment with doxorubicin and collected 5 days after senescence induction.** (**A, B**) Densitometric analysis of protein levels in HCT116 p53WT (**A**) and MCF-7 (**B**), n=9; statistical analysis was performed using paired one-tailed t-Student test. Relative protein expression means fold change (in the *expression* of proteins *relative* to the *expression* of GAPDH) vs appropriate control. Boxes: Q1, median, Q3; error bars: Minimum, Maximum. (**C**) Representative images from Western blotting of HCT116 p53WT and MCF-7 cell lysates. (**D, E**) Analysis of fluorescence intensity of anillin (whole nucleus area) and representative images (**F, G**) of control and doxorubicin-treated HCT116 p53WT (**D, F**) and MCF-7 cells (**E, G**), n=3; statistical analysis was performed using Wilcoxon matched-pairs signed-rank test. Data on graphs represent individual values for analyzed cells, median, error bars: Minimum, Maximum. Red – anillin, blue – DAPI stained DNA. Scale 20 µm. Statistical significance relative to control: ** p ≤ 0.01, *** p ≤ 0.001, **** p ≤ 0.0001

Senescence of two p53-proficient cancer cell lines, colon cancer (HCT116 p53WT) and breast cancer (MCF-7), was induced using doxorubicin (300 nM or 100 nM, respectively). The scheme of doxorubicin treatment was elaborated based on previous studies in which various models of senescence induction were developed for specific cancer cell lines [32, 35, 36]. The effectiveness of senescence induction was monitored by evaluating BrdU incorporation, SA-β-Gal activity and cell morphology (Supplementary Fig. 1) and by testing the level of selected proteins considered universal senescence markers (Fig. 2A, B, C). Anillin level was analyzed in both proliferating and senescent cells. We used the approach of 24 hours treatment followed by 5 days of culture in the fresh medium, as presented in Fig. 1. No spectacular differences in the effectiveness of senescence induction were observed when doxorubicin was applied for 5 days (Supplementary Fig. 2 and Supplementary Table 1). A significant decrease in lamin B1, HMGB1 and H3K9me3 (heterochromatin marker) was observed in MCF-7 and HCT116 p53WT cells treated with doxorubicin. The level of p53 protein was significantly higher in both cell lines after exposure to doxorubicin. Such changes and morphological alterations (Supplementary Fig. 3A and 4) confirm the model's validity for further studies. The increase in p53 was accompanied by an almost complete loss of anillin in both cancer cell lines. This observation suggests that the downregulation of anillin level is associated with cancer cell senescence and is inversely correlated with p53 level.

**Figure 3.**
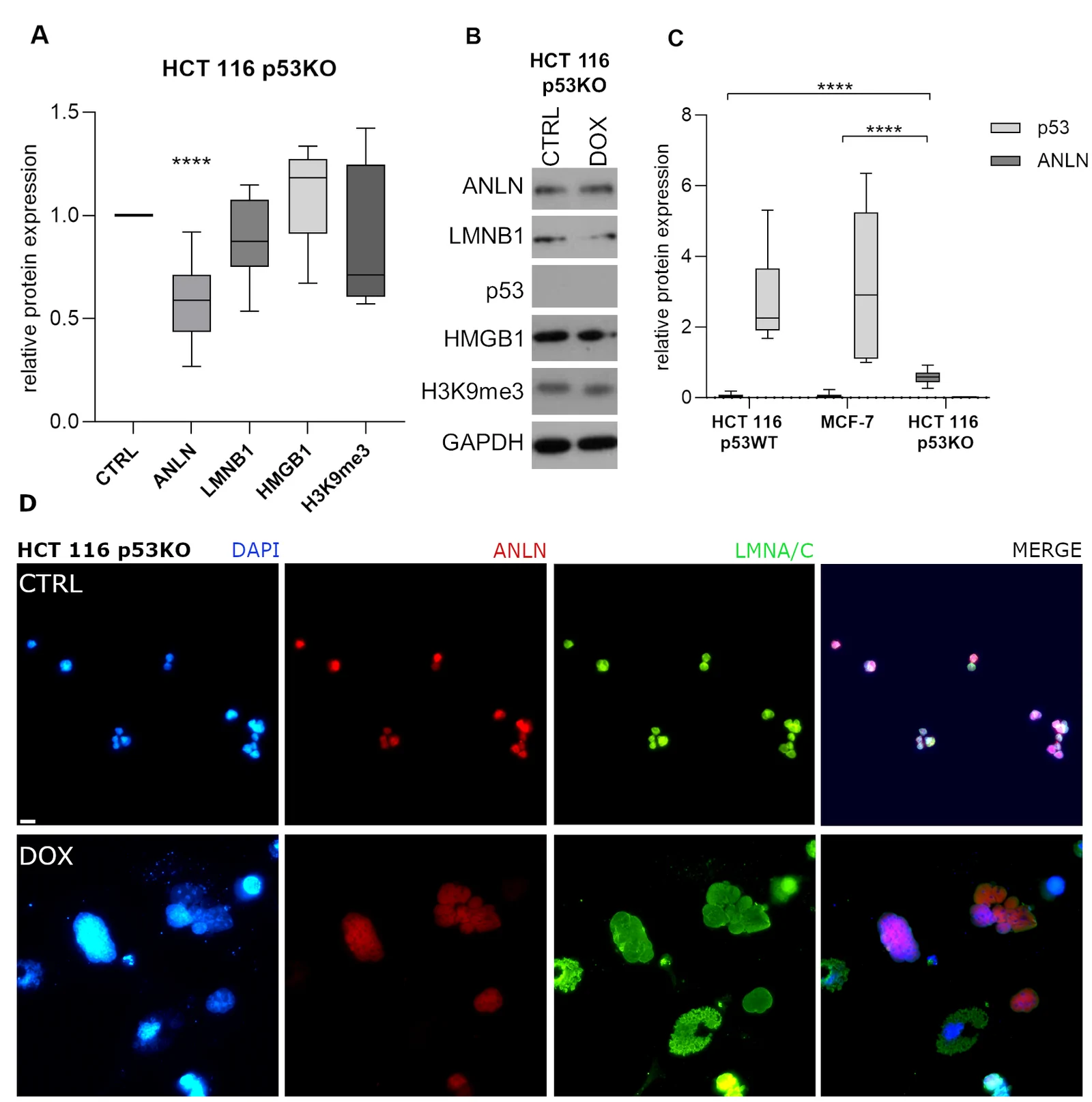
**Analysis of selected senescence markers and anillin levels in HCT 116 p53KO cells induced to senescence by doxorubicin treatment for 1 day and collected 5 days after senescence induction.** (**A**) Densitometric analysis of protein levels in control and doxorubicin-treated HCT116 p53KO based on Western blotting results, n=8; statistical analysis was performed using paired one-tailed t-Student test. Statistical significance is shown relative to control. (**B**) Representative images from Western blotting. (**C**) Comparison of anillin and p53 levels in p53-proficient (HCT116 p53WT and MCF7) and p53-deficient (HCT116 p53KO) cells treated with doxorubicin and analyzed using Western blotting, densitometric analysis (normalized to the level of anillin or p53 in control cells), n=3; statistical analysis was performed using Kruskal-Wallis test. Statistical significance is shown for differences between indicated cell lines. Relative protein expression means fold change (in the *expression* of proteins *relative* to the *expression* of GAPDH) vs appropriate control. (**D**) Representative images of immunostained control and doxorubicin-treated HCT116 p53KO cells. Red – anillin, green – lamin A/C, blue – DAPI stained DNA. Scale 20 µm. Boxes: Q1, median, Q3; error bars: Minimum, Maximum. Statistical significance: **** p ≤ 0.0001

In proliferating cancer cells, anillin is located in the nucleus. Although in cells undergoing senescence, the level of anillin decreases significantly, which has also been evidenced by significantly reduced fluorescence intensity (Fig. 2D, E), it can still be detected in the nucleus (Fig. 2F, G). Immuno-labelling of anillin revealed cell-type-dependent nucleolar localization of the protein in senescent cells. The nucleolar accumulation of anillin was observed only in MCF-7 cells undergoing senescence (Fig. 2G and Supplementary Fig. 5) and was detected in about 80% of senescent cells. It was not observed in proliferating cells. To confirm the nucleolar localization of anillin in MCF-7 cells, double-labelling was performed using antibodies detecting nucleophosmin (B23), a nucleolus marker (Supplementary Fig. 5).

#### Anillin expression is regulated by p53

The HCT116 cell line lacking a functional *TP53* gene was used to verify the hypothesis assuming negative regulation of anillin expression through p53. HCT116 p53KO cells were treated with doxorubicin (100 nM), and the same senescence markers were examined. We used lower doxorubicin concentration because that used in HCT116 p53WT, namely 300 nM, induced death rather than proliferation inhibition of HCT116 p53KO cells and caused pronounced nuclear deformation (Supplementary Fig. 6). The effect of 100 and 300 nM doxorubicin treatment was analyzed by cell counting and BrdU incorporation after 24 and 48 hours (Supplementary Fig. 3B) and by cell morphology (Supplementary Fig. 3A).

**Figure 4.**
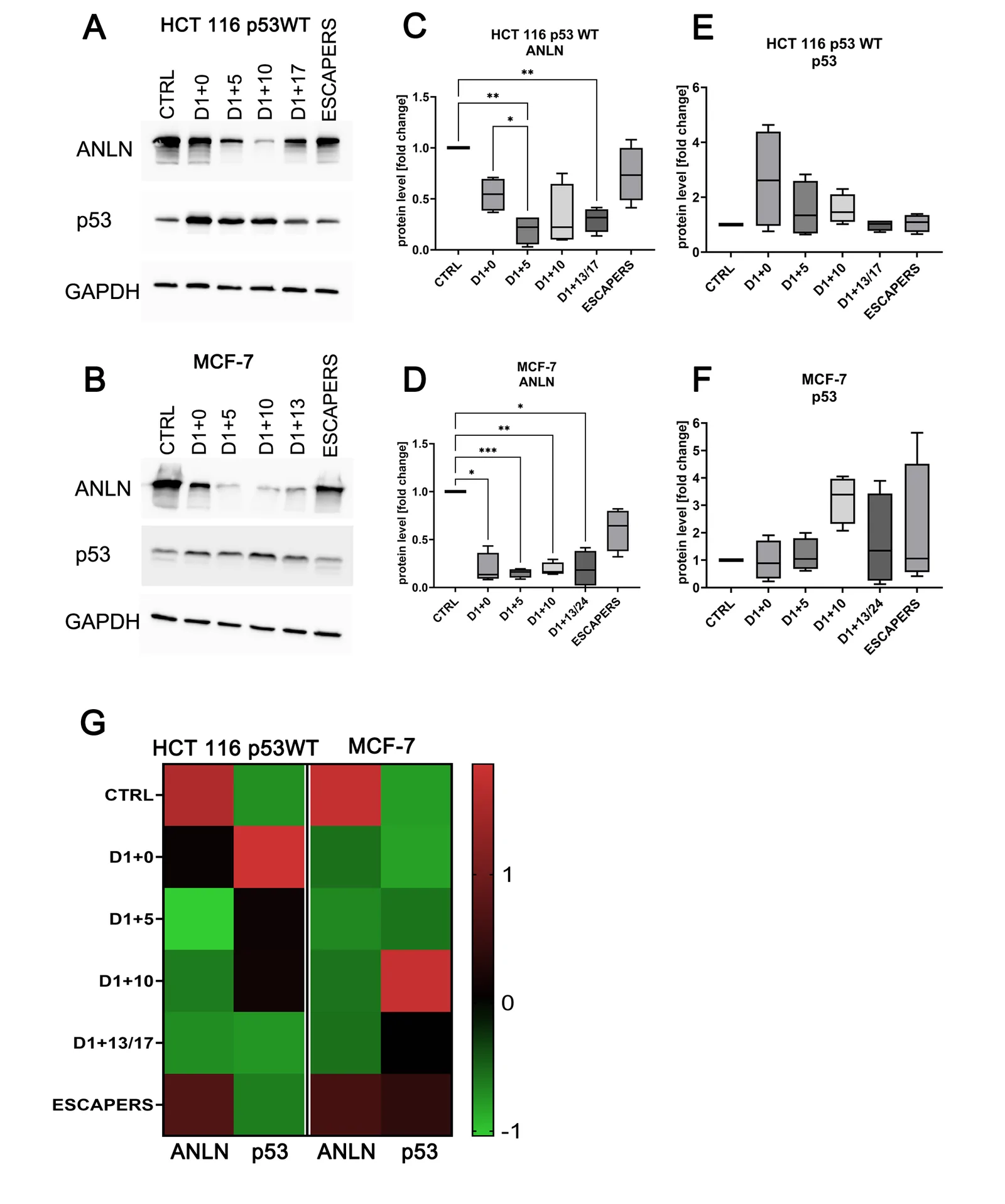
**Correlation between p53 and anillin levels during senescence and the escape from senescence in breast cancer MCF-7 and colon cancer HCT116 p53WT cells** (see Fig. 1). (**A-B**). Representative Western blots showing the levels of anillin and p53 in HCT116 p53WT cells (**A**) and MCF-7 cells (**B**). (**C-F**) The level of anillin and p53 on subsequent days of cell culture after senescence induction by doxorubicin in HCT116 p53WT (**C** and **E**) and MCF-7 cells (**D** and **F**) n = 4; statistical analysis was performed using one-way ANOVA followed by post hoc analysis (Tukey’s honest significant difference test; HSD test). Statistical significance of differences between indicated days of treatment: * p ≤ 0.05, ** p ≤ 0.01, *** p ≤ 0.001. Boxes: Q1, median, Q3; error bars: Minimum, Maximum. (**G**) The heat map shows the levels of anillin and p53 during senescence and escape from senescence in MCF-7 and HCT116 p53WT cells. Heatmap: Original data points are standardized into z-scores.

We analyzed senescence markers 5 days after doxorubicin administration (D1+5, Fig. 1). We observed that, despite proliferation arrest and morphological alterations and an increased number of cells with elevated activity of SA-β-Gal, HCT116 p53KO cells did not undergo classical senescence upon doxorubicin treatment (Fig. 3 and Supplementary Fig. 3A and 7). In particular, no decrease in lamin B1, HMGB1 and H3K9me3 levels was observed in these cells (Fig. 3A, B). However, the level of anillin decreased by about 40% compared to untreated cells. HCT116 p53KO cells treated with doxorubicin had higher levels of anillin than the analogous HCT116 p53WT cells (Fig. 3C). In HCT116 p53KO cells a nuclear localization of anillin was detected (Fig. 3D), in spite of the fact that the nucleus was very often deformed due to doxorubicin treatment (Supplementary Fig. 6). Lamin A/C labelling revealed the formation of numerous nuclear vesicles (nuclear blebbing). The structure of the nucleus made it impossible to identify the nuclear boundaries and evaluate the average fluorescence intensity of anillin in HCT116 p53KO cells. The results suggest that p53-deficient cells did not undergo classical senescence and anillin remained at relatively high level, supporting the hypothesis that p53 may be a negative regulator of anillin expression. In HCT116 p53KO cells, similarly to HCT116 p53WT cells, no nucleolar localization of anillin was observed after doxorubicin treatment. Thus, nucleolar localization seems to be cell-type specific and p53-independent. To decipher the meaning of this observation, additional studies are needed.

### Anillin appears in cells escaping from senescence when p53 levels gradually decline

It has been shown that senescence of HCT116 p53WT and MCF-7 cells is only transient and, after several days, cells resume division [37, 35, 38]. We have examined if the resumption of proliferation is related to anillin upregulation and if the latter is accompanied by a decrease in p53. To this end, cells were collected 5, 10, 13-24 days after doxorubicin treatment (300 nM for HCT116 p53WT and 100 nM for MCF-7 cells). Escape from senescence was documented by decreased SA-β-Gal activity and an increase in BrdU-positive cells (Supplementary Fig. 1).

We determined that anillin gradually reappeared in tumor cells escaping from senescence (Fig. 4A-D) and that this was correlated with gradual downregulation of p53 (Fig. 4A, B, E, F). In both cell lines, MCF-7 and HCT116 p53WT, an almost complete decay of anillin was observed on days 5 and 10, and its reappearance between days 13 and 17 (Fig. 4C, D). However, in both cell lines, the anillin level was still significantly lower than in control, untreated cells. Anillin reappeared only in escapers, a population of proliferating cells derived from senescent cells. The level of p53 slightly increased during senescence development and decreased during senescence escaping to the level observed in control, proliferating cells. The results show that anillin is reexpressed after the cells resume proliferation as a result of senescence escaping. This is associated with the gradual decrease in p53 (the effect is more pronounced in HCT116 p53WT cells), which supports the role of p53 in regulating anillin expression. The summary of the obtained results showing an inverse correlation between p53 and anillin level during senescence development and escape is given as a heat map (Fig. 3G).

## DISCUSSION

Our studies have shown that anillin was strongly downregulated in senescent cancer cells. We observed, however, that in p53-deficient cells the level of anillin dropped only slightly. It should be underlined that p53-proficient cells underwent senescence exhibiting numerous classical markers, including a significant increase in p53 level. On the contrary, p53-deficient cells, even though they stopped to proliferate and had altered morphology, did not express other typical symptoms of senescence after doxorubicin treatment. Previous studies have shown that HCT116 p53KO cells could undergo senescence after continuous treatment with doxorubicin (100 nM, 5 days) but with lower efficiency, for example, a smaller percentage of cells enter the senescence displayed the increased activity of SA-β-Gal (40% compared to 90% of HCT116 p53WT cells) [39]. The senescence of cancer cells due to chemotherapeutics has been well documented [32, 40, 41]. However, it has also been shown that senescence of cancer cells can be reversible and that cells resume division sometime after drug treatment, which can be associated with tumor regrowth.

**Figure 5.**
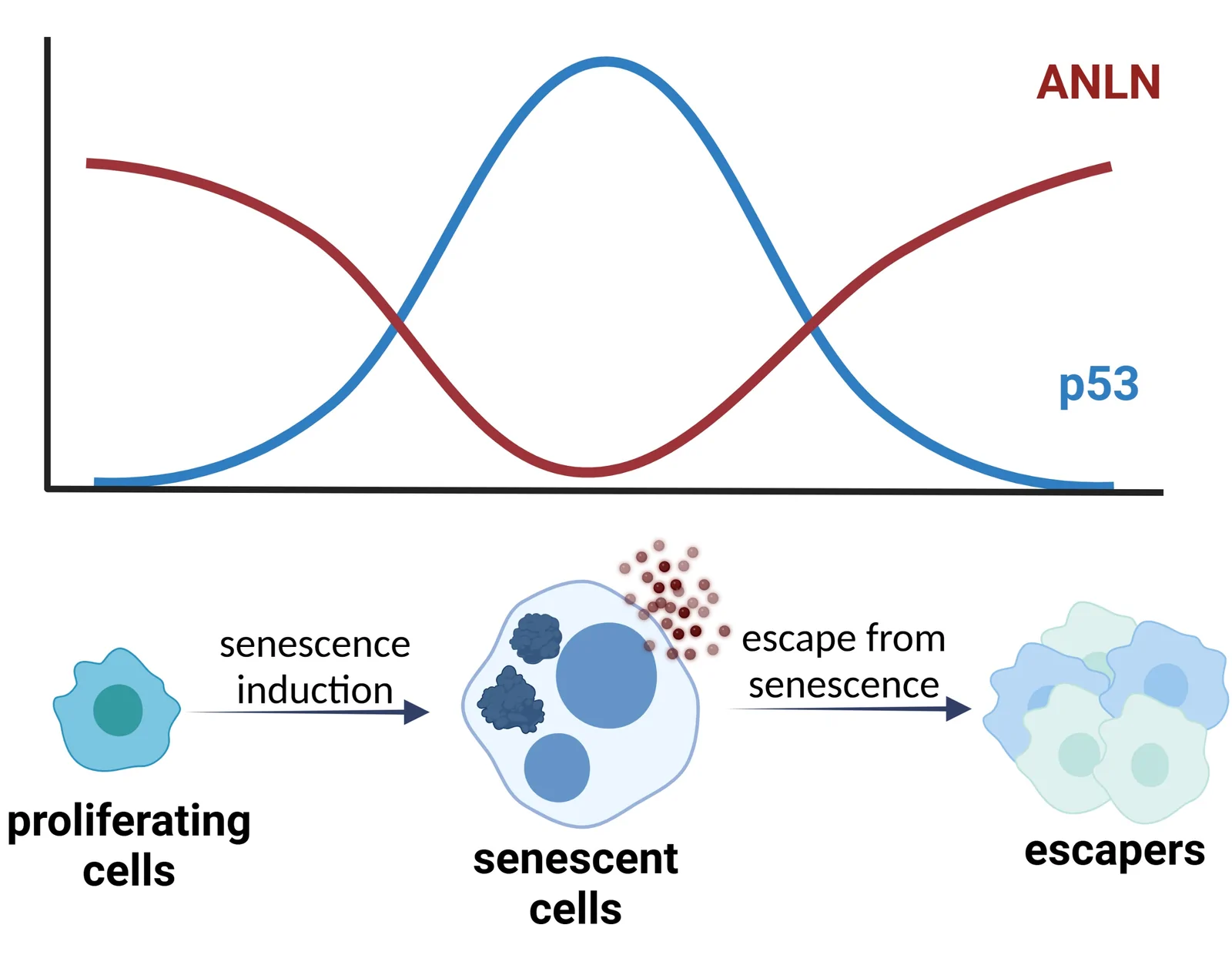
**Inverse correlation between ANLN and p53 during senescence and escape from senescence in cancer cells.** Downregulation of anillin is a consequence of the induction of p53 due to cellular senescence. After some time, senescent cells resume divisions, which is associated with a decrease in p53 levels and an increase in anillin. Performed with Biorender.

We proposed anillin as a helpful marker of proliferating cells (similar to Ki67) and decreased anillin level as a potential additional senescence marker. The expression pattern of anillin seems similar to that of the Ki67 protein, which is commonly used to detect proliferation-competent cells and is considered a prognostic marker of tumor aggressivity. Linear dependence between Ki67 and anillin has been postulated [11], but other researchers claim that anillin is a Ki67-independent marker [22]. It is postulated that enhanced expression of anillin is probably not a simple consequence of being a division marker. To this date, there are no data concerning the function and changes in anillin level in senescent normal or tumor cells. However, it has been shown that anillin silencing led to senescence of cancer cells [22]. The cells became large and polyploid. It remains an open question whether senescence of cancer cells induced by anillin knockdown is permanent or transient and whether resumption of anillin expression can promote senescence escape and cancer relapse. In our study, senescence was associated with a decrease in anillin, and in the paper mentioned above, the loss of anillin was sufficient for senescence induction. It is also unclear if the loss of anillin is indispensable for senescence induction. HCT116 p53KO cells treated with doxorubicin exhibited only certain senescence markers and also in a smaller percentage of cells, but this could be related to the lack of p53 and not the higher level of anillin. Neither do we suppose that anillin reappearance is crucial for escaping from senescence. Future studies are needed to determine this.

Based on weighted gene co-expression network analysis (WGCNA), a downregulation of anillin gene expression in senescent fibroblasts has already been reported [42]. We have also observed that anillin's gene expression and protein level decreased in normal cells (VSMC, fibroblasts, preadipocytes) undergoing senescence (manuscript in preparation). Now, we have observed a similar phenomenon in cancer cells. Anillin is a good marker for determining tumor cells escaping from senescence because, as our study has shown, its reappearance is associated with proliferation resumption. We have also shown that the level of anillin seems to be regulated by p53. p53 is the main regulator of senescence and is activated in both DNA damage-dependent and DNA-damage-independent senescence [43, 44]. This protein stops the cell cycle and prevents replication of potentially leading to tumor transformation cells with damaged genetic material. Moreover, the p53 protein is a transcription factor for an extensive group of genes [25]. Cancer cells lacking functional p53 protein due to mutations or a complete loss of p53 expression give rise to more aggressive tumors and show lower drug sensitivity [45-47]. Since anillin is involved in cytokinesis, it is commonly considered only a marker of proliferation. However, its presence in the nucleus of postmitotic cells suggests a function different than that in cell division. Nuclear localization of anillin was detected in this study and previous ones [10-11]. In our studies, anillin was observed in both untreated cells and cells induced to senesce. Since anillin has an actin-binding domain, it can be assumed that both proteins participate in regulating processes occurring in the nucleus, as it was already reported based on large-scale studies [14]. Nuclear actin plays an important role in modulating chromatin structure since it is a component of chromatin remodeling complexes and preinitiation complexes, to which RNA polymerases are recruited [16, 48]. Actin influences transcription and, thus, gene expression, while anillin, as a protein interacting with actin, may contribute to this regulation.

The nucleolar localization of anillin was observed only in MCF-7 cells induced to senesce. So far, such property has not been observed in any other type of senescence of normal cells analyzed by us or in HCT116 cancer cell lines. The nucleolar localization of anillin was not p53-dependent because this phenomenon was not characteristic of p53-proficient HCT116 cells. The reason behind the accumulation of anillin in the nucleoli requires further research.

Anillin upregulation was observed in many cancer cell types, and it was related to more aggressive cancer progression. It has been postulated that in certain types of cancer, anillin can be used as a prognostic marker. Considering that cancer cells very often have mutated p53 (unfunctional or deleted), the lack of that vital negative regulator of anillin could be responsible for its upregulation in p53-deficient cancer cells. However, to date, there is no data showing if the level of anillin in tumor cells with a wild-type copy of p53 is lower than in cells with unfunctional p53.

Although in cells with functional p53, HCT116 and MCF-7, doxorubicin has induced typical symptoms of senescence, the process was reversible since cells resumed divisions after about 10-17 days. Long-term observations have shown that anillin levels gradually increased in cells that have resumed divisions, while that of p53 decreased. Because the p53-deficient HCT116 cells did not undergo classical senescence, the DNA damage caused by doxorubicin can probably direct cells to undergo cell death instead of senescence. HCT116 p53KO cells exhibited significantly pronounced morphological changes and nuclear disorders, and studies conducted so far have shown a lower effectiveness of HCT116 p53KO cells in undergoing senescence. Therefore, HCT116 p53KO was not used in studies concerning escape from senescence [49].

Negative regulation of anillin expression by p53 has been postulated in the literature [20, 26, 27]. It has been proposed that enhanced anillin expression in cancer cells may be the consequence of p53 deficiency. However, these were mainly *in silico* studies and none was related to senescence. Few studies, performed using chromatin immunoprecipitation combined with sequencing, provide experimental evidence for such regulation. Our experimental data obtained using two cell lines differing only in p53 status has shown that this protein negatively regulates anillin and could be the reason for downregulation of anillin expression during senescence. However, in p53-deficient cells, a partial decrease of anillin was also detected, suggesting a more complicated mechanism regulating anillin expression.

It cannot be ruled out that not only does p53 regulate the expression of anillin, but anillin also somehow affects the p53 level. It was observed that silencing of anillin caused polyploidization of cancer cells, and their further proliferation was inhibited by p53 [50]. Our data also suggest a negative feedback between anillin and p53, although it may as well be the result of a stress response. Further studies are needed to solve this issue.

## Conclusions

We have shown for the first time that the senescence of cancer cells is associated with anillin downregulation and that the escape from senescence is related to anillin upregulation. This demonstrates that a decrease in anillin level accompanies senescence, regardless of whether it is permanent (normal senescent cells) or transient (tumor cells escaping from senescence). However, this refers only to p53-dependent senescence. Senescence-associated anillin downregulation could be related to its role in cytokinesis, and therefore, its synthesis was renewed when cells resumed division, as shown in cells escaping from senescence. However, considering the presence of anillin in the nucleus, a certain division-independent role can be assumed. We have proved that p53 is a negative regulator of anillin, and the downregulation of anillin is correlated with p53 upregulation, which is a vital feature of senescence. A summary of the results is presented in Figure 5.

## Supplementary Materials

The Supplementary data can be found online at: www.aginganddisease.org/EN/10.14336/AD.2025.0402.

## Data Availability

The datasets generated during and/or analyzed during the current study are available from the corresponding author upon reasonable request.
