# Supplementary material for: Anillin Recedes in p53-Dependent Senescence of Tumor Cells and Reappears in Cells Escaping from Senescence
Source: Aging Dis. 2025 May 10;17(3):1590–603. doi: 10.14336/AD.2025.0402 (PMC13061550; doi:10.14336/AD.2025.0402)
Supplement: Supplementary file 1 — The Supplementary data can be found online at: www.aginganddisease.org/EN/10.14336/AD.2025.0402. [file AD-17-3-1590-s.pdf]

SUPPLEMENTARY DATA

Anillin Recedes in p53-Dependent Senescence of Tumor Cells and Reappears in Cells Escaping from Senescence

Tomasz Buko, Karolina Staniak, Magdalena Dudkowska, Dorota Janiszewska, Dominika Dębowska, Agnieszka Gadecka, Anna Bielak-Zmijewska

© 2025. Buko T et al. Published online at http://www.aginganddisease.org/EN/10.14336/AD.2005.0402

SUPPLEMENTARY DATA

Supplementary Table

Supplementary Table 1. Comparison of two experimental approaches (D1+5 and 5D) inducing cell senescence. D1+5 – 1-day treatment with doxorubicin and then culture for 5 days in fresh medium. 5D – treatment with doxorubicin for 5 days (diagrams below the table show the experimental kinetics). Statistical analysis was performed using a one-tailed t-Student test. Statistical significance (relative to control): *** p ≤ 0.001, **** p ≤ 0.0001

cell types	analysis	**D1+5** [% of positive cells]	**5D** [% of positive cells]
MCF-7	SA-β-Gal	**63.75** ±9.98 p=0.0004 ***	**83.98**±7.84 p<0.0001 ****
	BrdU	**14.30**±4.37 p<0.0001 ****	**8.30**±0.10 p<0.0001 ****
HCT116 p53WT	SA-β-Gal	**80.75**±7.18 p<0.0001 ****	**61.92**±4.58 p<0.0001 ****
	BrdU	**24.45**±6.61 p=0.001 ***	**14.86**±3.70 p<0.0001 ****
HCT116 p53KO	SA-β-Gal	**36.49**±12.01 p=0.065 NS	**47.23**±7.23 p=0.0004 ***
	BrdU	**50.10**±6.01p=0.0003 ***	**43.41**±4.63 p<0.0001 ****

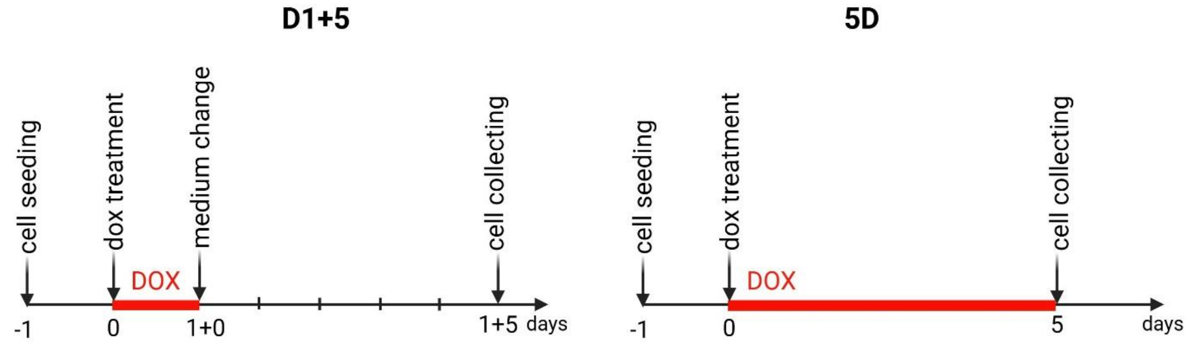

© 2025. Buko T et al. Published online at http://www.aginganddisease.org/EN/10.14336/AD.2005.0402

SUPPLEMENTARY DATA

Supplementary Figures

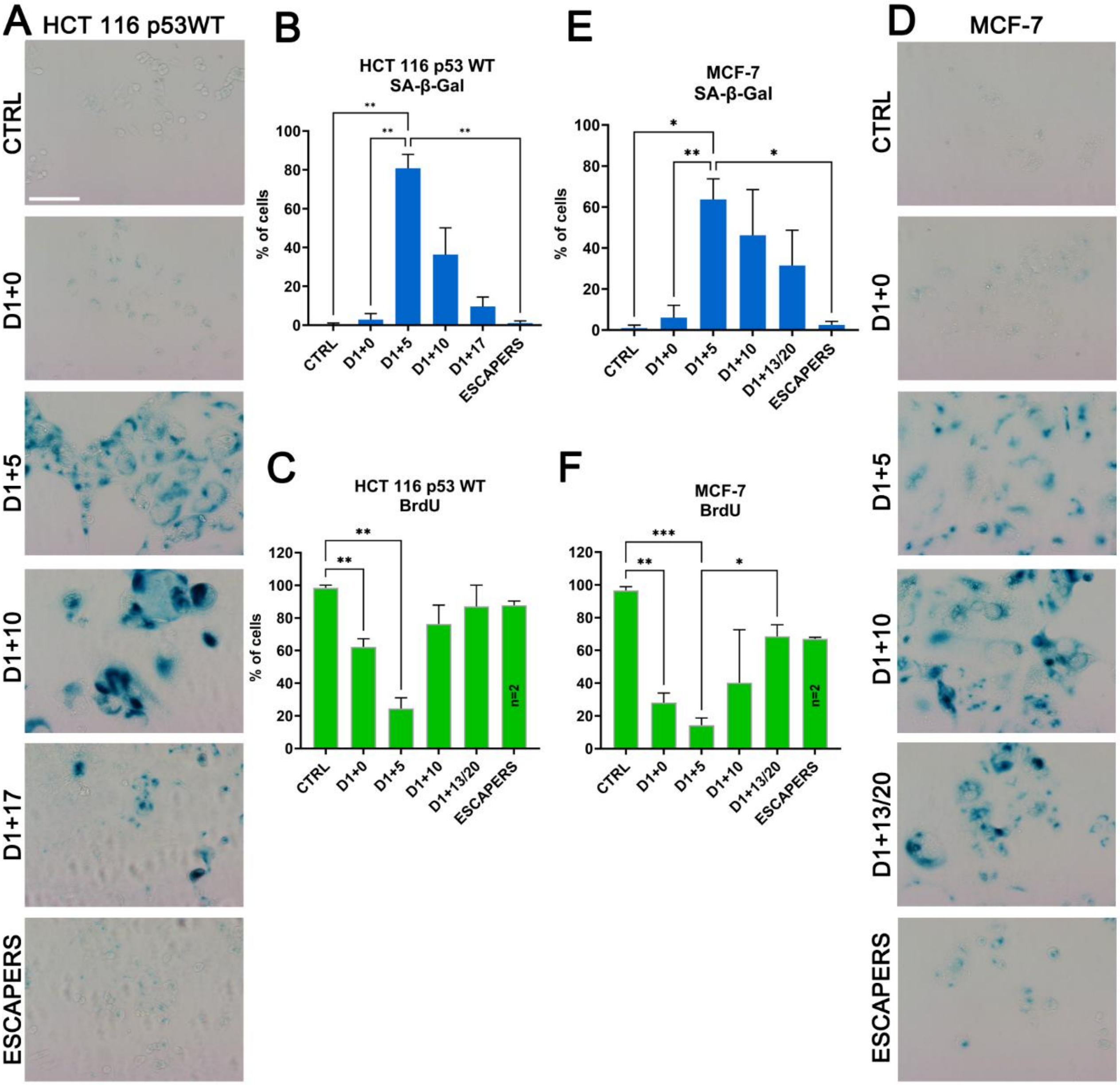

Supplementary Figure 1. Senescence efficiency assessment in HCT p53WT and MCF-7 cells treated for 1 day with doxorubicin (100 and 300 nM, respectively) and then cultured in fresh medium (see Fig. 1). The activity of SA-β-Gal and BrdU incorporation during senescence and the escape from senescence. (**A**) Representative phase contrast images (blue staining represents SA-β-Gal activity) in HCT116 p53WT cells. (**B**) Quantitative analysis of the number of SA-β-Gal-positive HCT116 p53WT cells. (**C**) Quantitative analysis of the number of BrdU-positive HCT116 p53WT cells. (**D**) Representative phase contrast images (blue staining represents SA-β-Gal activity) in MCF-7 cells. (**E**) Quantitative analysis of the number of SA-β-Gal -positive MCF-7 cells. (**F**) Quantitative analysis of the number of BrdU-positive MCF-7 cells. Data were calculated as the percentage of the total cell population and presented as means ± SD. n=4 except when indicated otherwise. Statistical analysis was performed using one-way ANOVA followed by post hoc analysis (Tukey's honest significant difference test; HSD test). Statistical significance of differences between subsequent days of treatment: * $p \leqslant 0.05$, ** $p \leqslant 0.01$, *** $p \leqslant 0.001$. Scale – 100 µm

SUPPLEMENTARY DATA

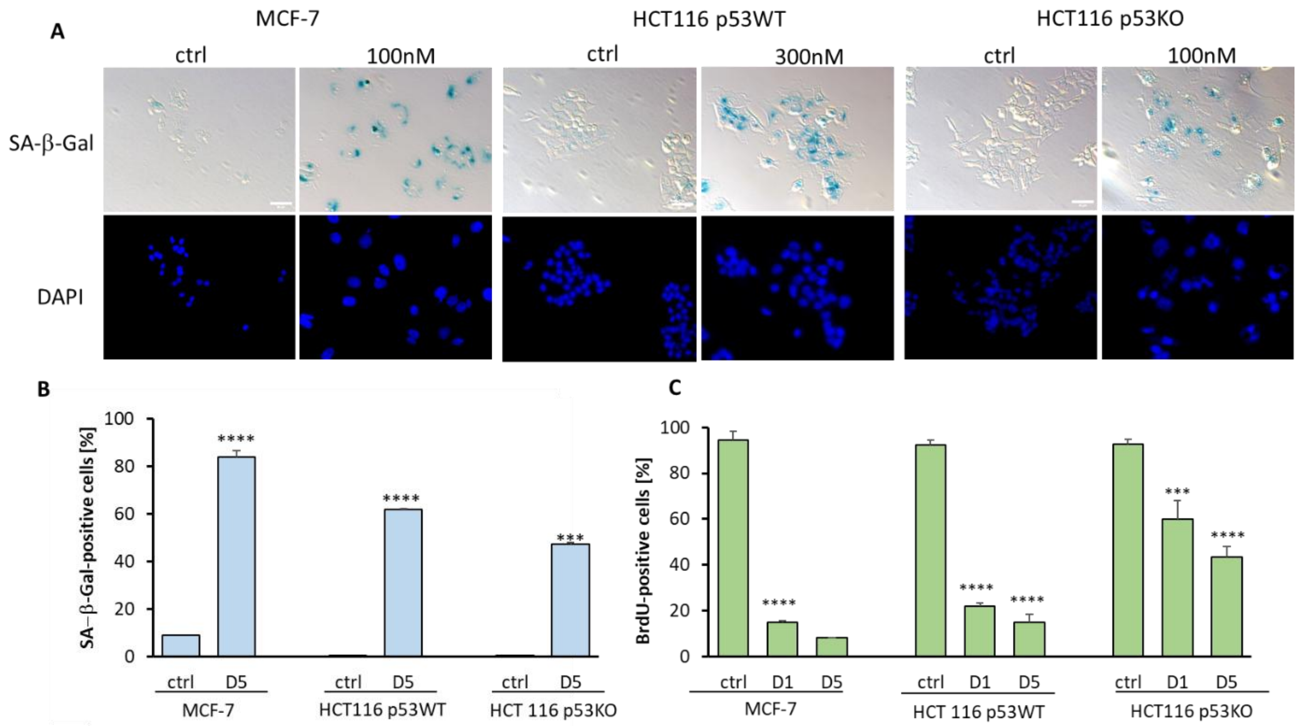

Supplementary Figure 2. Senescence efficiency assessment in MCF-7, HCT p53WT and HCT p53KO cells treated 5 days with doxorubicin (100, 300, 100 nM, respectively). Analysis of the activity of SA-β-Gal and BrdU incorporation during senescence induction. (**A**) Representative phase contrast (blue staining represents SA-β-Gal activity) and fluorescent microscope (nuclei were stained with DAPI) images of control and treated cells. (**B**) Quantitative analysis of the number of SA-β-Gal -positive cells. (**C**) Quantitative analysis of the number of BrdU-positive cells. Data were calculated as the percentage of the total cell population and presented as means ± SD. n=3; statistical analysis was performed using a paired one-tailed t-Student test. Statistical significance relative to control: *** $p \leqslant 0.001$, **** $p \leqslant 0.0001$. Scale – 50 μm.

© 2025. Buko T et al. Published online at http://www.aginganddisease.org/EN/10.14336/AD.2005.0402

SUPPLEMENTARY DATA

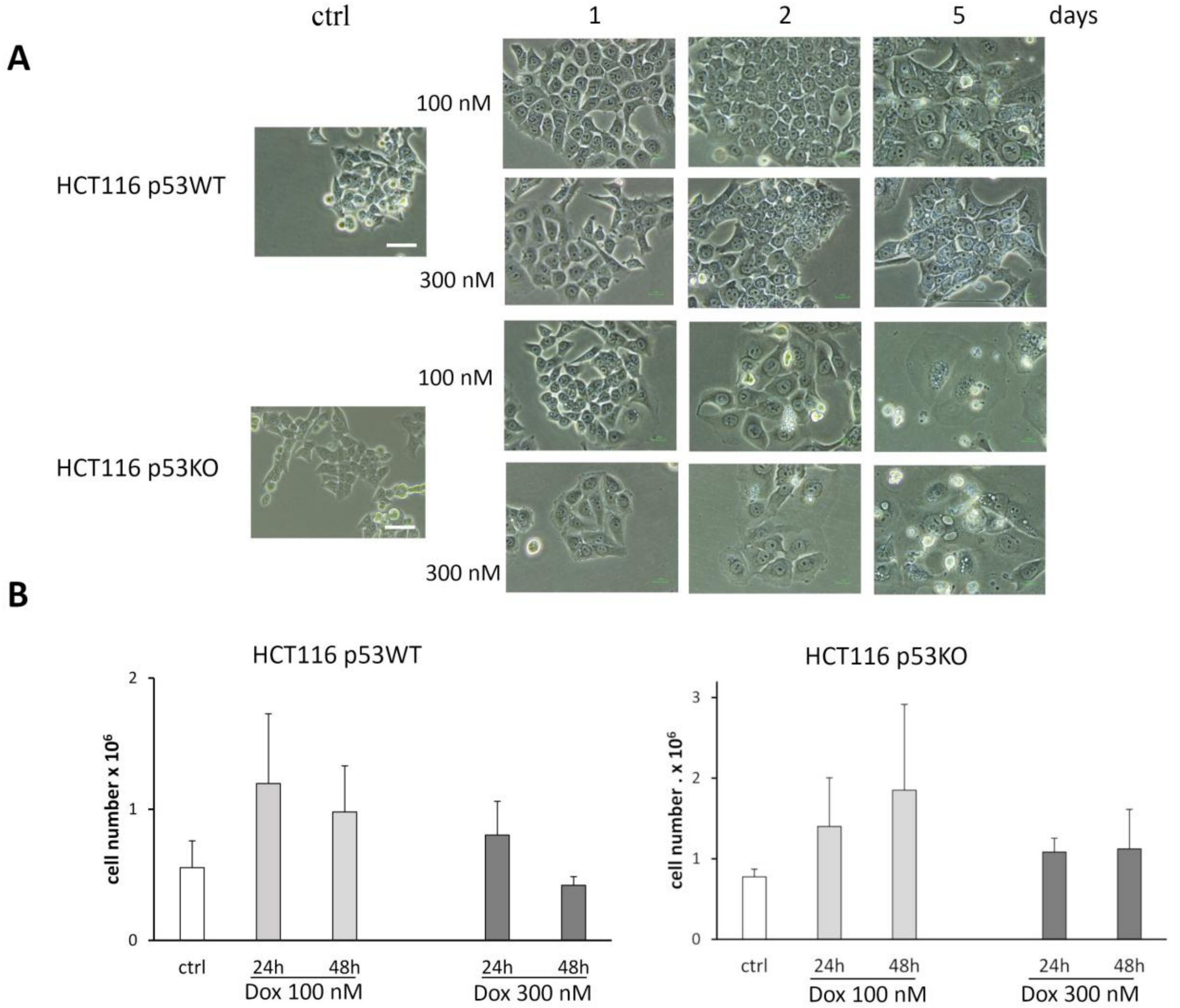

Supplementary Figure 3. Selection of senescence-inducing concentration of doxorubicin (100 vs. 300 nM, permanent treatment without changing the medium) in HCT116 p53WT and HCT116 p53KO cells. Analysis of short-time treatment -24 and 48 hours. (**B**) cell counting (**A**) morphological changes – representative phase contrast images. No statistical significance. n=3; statistical analysis was performed using a paired one-tailed t-Student test. Scale – 50 µm. For HCT116 p53KO cells, the 100 nM concentration was efficient in stopping proliferation and inducing morphological changes; 300 nM concentration induced cell death rather than senescence, as evidenced by decreased number of cells (B) and the presence of floating cells in the culture **(A**). In the case of HCT116 p53WT, 100 nM doxorubicin did not stop proliferation (**B);** therefore, 300 nM concentration was used.

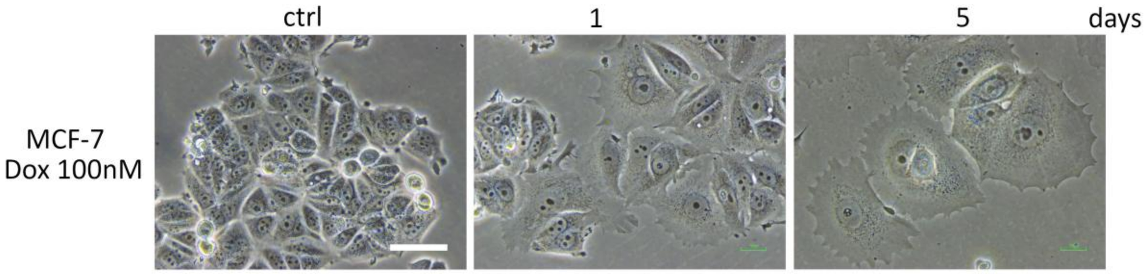

Supplementary Figure 4. Morphological changes in MCF-7 cells induced to senesce with doxorubicin treatment (100 nM, permanent treatment without changing the medium). Representative phase contrast images. Scale – 50 µm.

© 2025. Buko T et al. Published online at http://www.aginganddisease.org/EN/10.14336/AD.2005.0402

SUPPLEMENTARY DATA

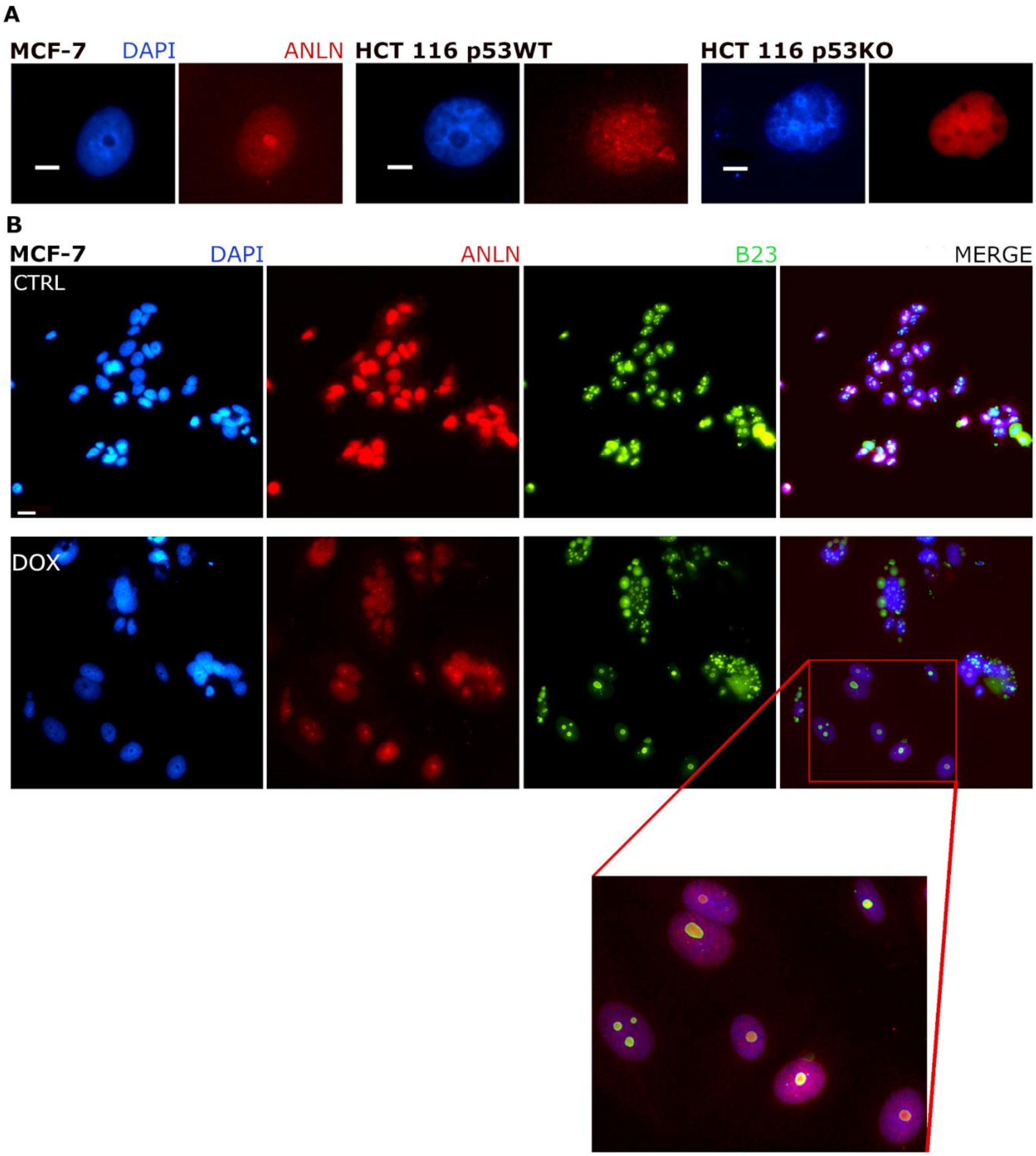

Supplementary Figure 5. Detection of anillin in the nucleoli of senescent cells (cells treated for 1 day with doxorubicin and then cultured in a fresh medium, D1+5). (**A**) representative images of MCF-7, HCT116 p53WT and HCT116 p53KO cells. Scale 10 µm (**B**) MCF-7 cells. Scale 20 µm. Blue – DAPI stained nucleus, red – anillin, green – B23 (nucleophosmin, a nucleolus marker)

© 2025. Buko T et al. Published online at http://www.aginganddisease.org/EN/10.14336/AD.2005.0402

SUPPLEMENTARY DATA

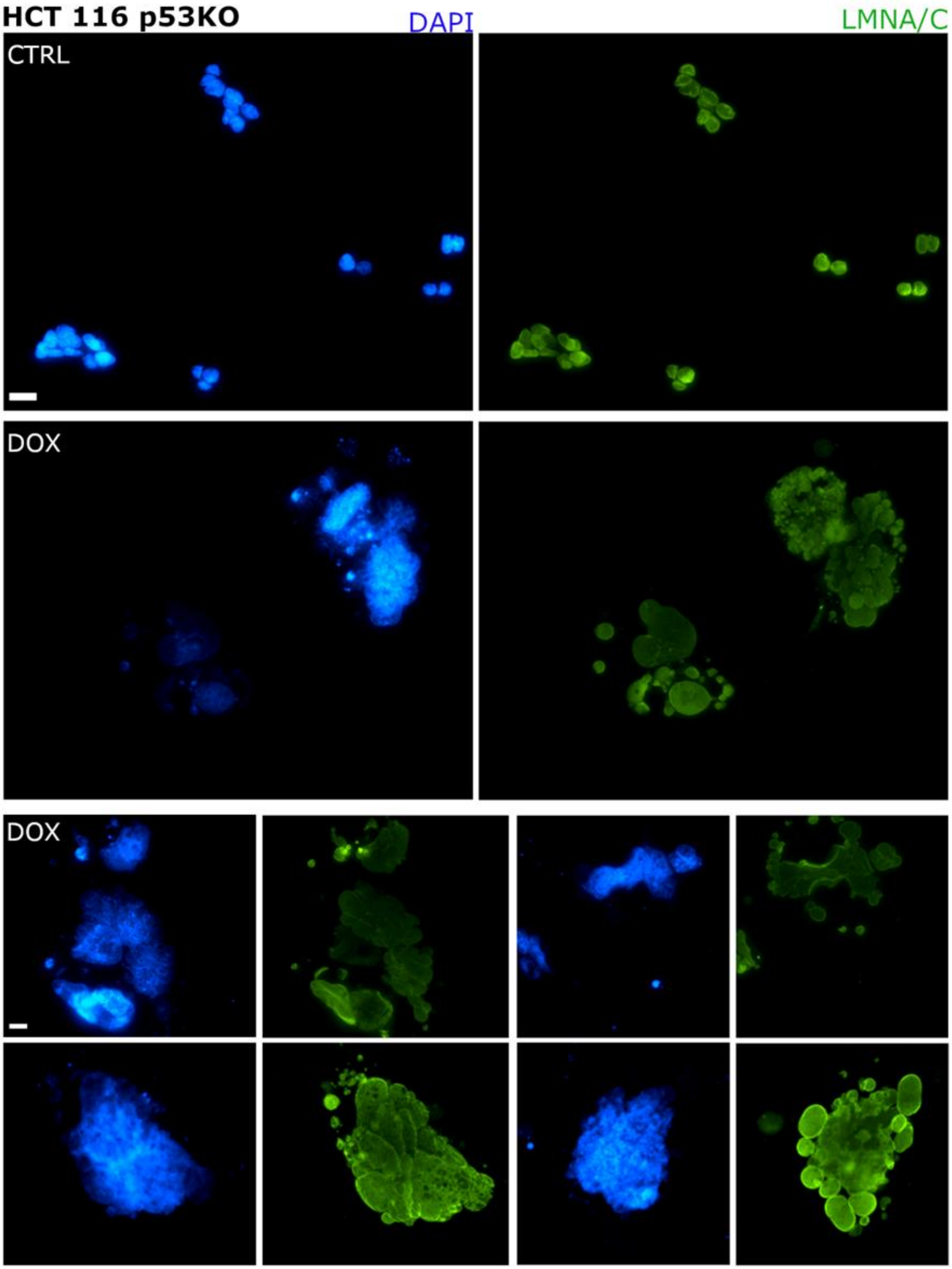

Supplementary Figure 6. Nucleus deformation in HCT116 p53KO cells treated with 100 nM doxorubicin for 1 day and analyzed after 5 days of culture in a fresh medium. Representative fluorescent microscope images of control and treated cells. Green – lamin A/C, blue – DAPI stained DNA. Scale – 20 μm

© 2025. Buko T et al. Published online at http://www.aginganddisease.org/EN/10.14336/AD.2005.0402

SUPPLEMENTARY DATA

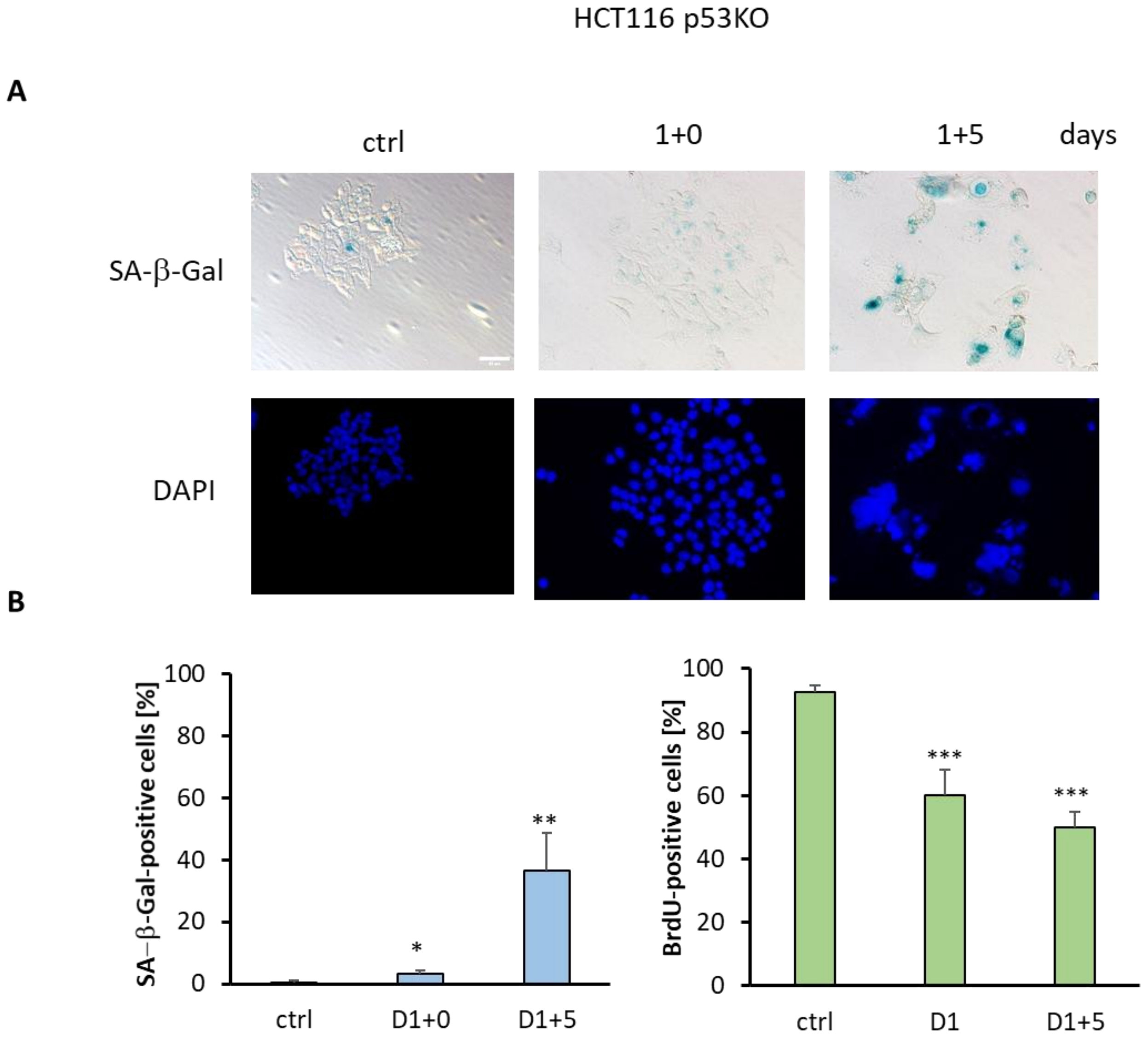

Supplementary Figure 7. Senescence efficiency assessment in HCT116 p53KO cells, treated for 1 day with doxorubicin (100 nM) and then cultured in fresh medium, based on SA-β-Gal activity and BrdU incorporation. (**A**) Representative phase contrast (blue staining represents SA-β-Gal activity) and fluorescent microscope (nuclei were stained with DAPI) images of control and treated cells. (**B**) Quantitative analysis of the number of SA-β-Gal -positive cells. (**C**) Quantitative analysis of the number of BrdU-positive cells. Data were calculated as the percentage of the total cell population and presented as means ± SD. n=3; statistical analysis was performed using a paired one-tailed t-Student test. Statistical significance relative to control: * $p \leqslant 0.05$, ** $p \leqslant 0.01$, *** $p \leqslant 0.001$. Scale – 50 µm.

© 2025. Buko T et al. Published online at http://www.aginganddisease.org/EN/10.14336/AD.2005.0402
